# Effectiveness and cost of quick diagnostic tests to determine tetanus immunity in patients with a wound in french emergency departments

**DOI:** 10.1186/s12879-014-0603-3

**Published:** 2014-11-19

**Authors:** Dieynaba S N'Diaye, Michaël Schwarzinger, Dorothée Obach, Julien Poissy, Sophie Matheron, Enrique Casalino, Yazdan Yazdanpanah

**Affiliations:** INSERM, IAME, UMR 1137, Paris, F-75018 France; Université Paris Diderot, IAME, UMR 1137, Sorbonne Paris Cité, Paris, F-75018 France; UPMC Université Paris 06, Paris, ED393, F-75005 France; Centre Hospitalier et Universitaire de Lille, Service d’Urgence Respiratoire, Réanimation Médicale et Médecine Hyperbare, Université de Lille II, Lille, France; AP-HP, Hôpital Bichat, Service de Maladies Infectieuses, Paris, F-75018 France; AP-HP, Hôpital Bichat, Service des Urgences, Paris, F-75018 France

**Keywords:** Tetanus immunity, Diagnostic tests, Cost-effectiveness, Emergency department

## Abstract

**Background:**

Tétanos Quick Stick® (TQS) is a test for tetanus immunity screening for wounded patients in emergency departments (EDs), but represents additional costs compared with a medical interview on vaccination history. The study objective was to assess the effectiveness and cost of the TQS in French EDs.

**Methods:**

We performed a model-based analysis that simulates screening of tetanus immunity and risk of tetanus based on prophylaxis administration. Strategies compared were: **i)** diagnosis of tetanus immunity by “TQS”; **ii)** “Medical Interview” (current practice). The study population was 1,658,000 French adults seeking ED care for a wound in 2012. Model parameters were estimated based on French national surveillance data, and published literature. Outcome measures were number of tetanus cases, life years gained and costs (2012 €) from a societal perspective.

**Results:**

Use of TQS had negligible impact on health outcomes (0.02 tetanus cases/year in France vs. 0.41 for “Medical Interview”), but resulted in a decrease in annual costs of €2,203,000 (-42%). Base case and sub-group analysis showed that with the same effectiveness, the average cost per patient was: €13 with “Medical Interview” vs. €11.7 with TQS for the overall cohort; €28.9 with “Medical Interview” vs. €21 with “TQS” for tetanus-prone wounds; €15 with “Medical Interview” vs. €14.1 with “TQS” for patients aged ≥65 years; and €6.2 with “Medical Interview” vs. €7.8 with “TQS” for non-tetanus-prone wounds.

**Conclusions:**

Use of TQS is as effective and less costly than “Medical Interview” when applied in ED to wounded patients with tetanus-prone wounds or aged ≥65 years. However, it is more expensive in patients with non-tetanus-prone wounds.

**Electronic supplementary material:**

The online version of this article (doi:10.1186/s12879-014-0603-3) contains supplementary material, which is available to authorized users.

## Background

In high-income countries, thanks to systematic immunization campaigns and prophylaxis strategies, tetanus is a rare disease. In France, the annual incidence rate of tetanus for the period 2000-2012 is estimated to be 0.27 cases per million inhabitants [1].

Alongside universal vaccination, unscheduled tetanus prophylaxis is administered to patients with a wound depending on its severity and the patient's vaccination status (Table 1) [2],[3]. Currently, in emergency departments (EDs), healthcare workers assess tetanus immunization status by asking for the patient's vaccination card or through a medical interview retracing the patient's vaccination history. However, patients rarely bring their vaccination cards, and multiple studies have shown that medical interviews lack accuracy [4]-[8]. For example, Colombet et al. reported that the sensitivity and specificity of this practice was 62% and 79%, respectively [5].

**Table 1:** **Guidelines for tetanus prophylaxis for wound management in French EDs**

Type of wound	Vaccination history		
	Complete primary vaccination		No or unknown complete primary vaccination
	Booster ^a^updated ^b^	Booster not updated	
Non-tetanus-prone wound	Nothing	Booster	Booster (proposal to update the primary vaccination)^d^
Tetanus-prone wound^c^	Nothing	Booster + TIG	Booster + TIG (proposal to update the primary vaccination)

ED: Emergency department; TIG: Human tetanus immunoglobulins;

^a^Tetanus-toxoid vaccine.

^b^According to the French vaccination schedule; i.e. having received a tetanus-toxoid injection during the last 20 years for those <65 years of ageand during the last 10 years of those aged ≥65 years.

^c^Such as (but not limited to) wounds contaminated with dirt, feces, soil, and saliva; puncture wounds; avulsions; and wounds resulting from missiles, crushing, burns, and frostbite.

^d^Primary vaccination update: administration of two other boosters at a one-month interval, usually by a general practitioner.

Immunization status, as defined by serum tetanus antitoxin level, can now be more accurately determined through rapid testing using immunochromatographic methods. Colombet et al. recently showed that one of these tests, the Tétanos Quick Stick® TQS) (TQS; Ingen, France), was associated with a higher sensitivity and specificity than medical interview for immunization status determination [5]. However, such tests are associated with higher costs than the medical interview. The estimated unit cost of a test is €4.7 [9].

The objective of this study was, in the current context of implemented guidelines, to assess using a model-based analysis, in French patients presenting to the ED with wounds, the effectiveness, costs and cost-effectiveness of rapid tests and in particular TQS for tetanus immunization status determination vs. current practice based on the medical interview.

## Methods

### Analytic overview

**Figure 1:**
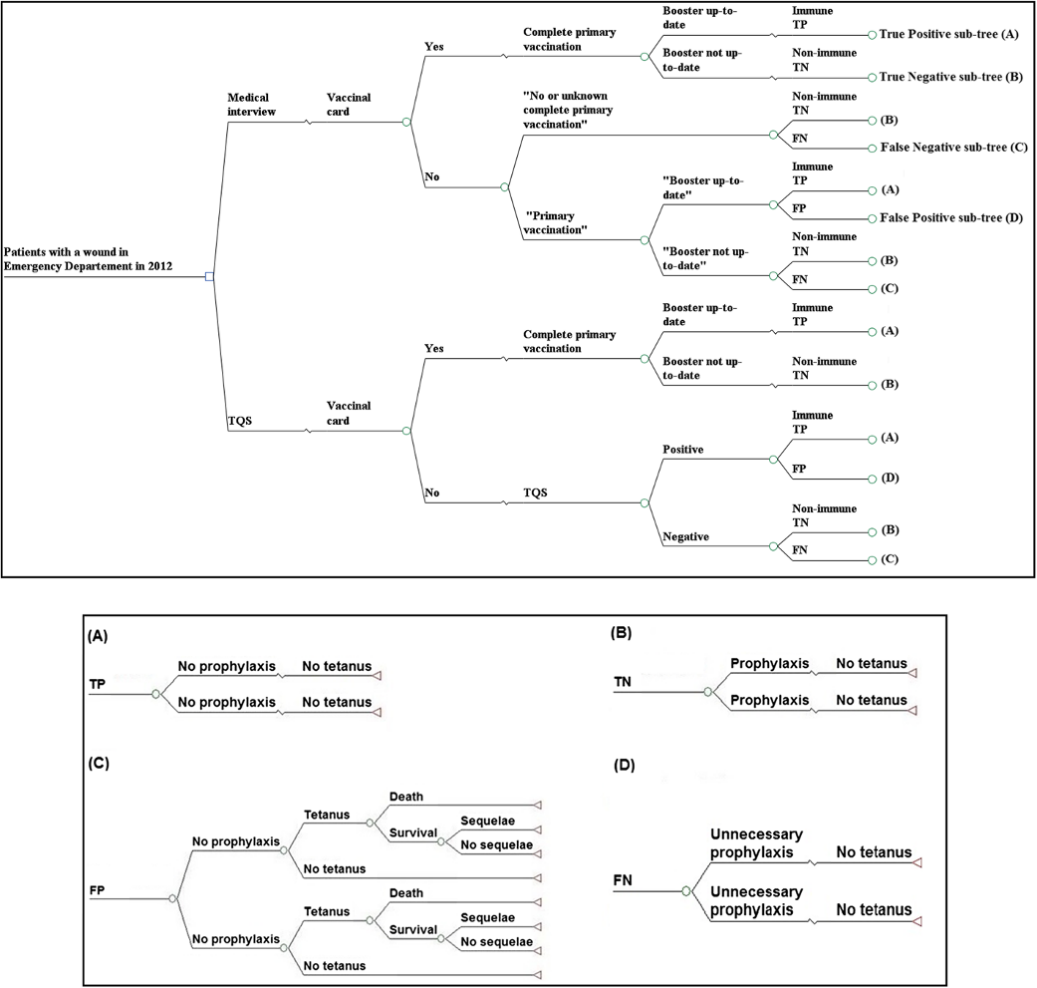
**Decision-tree model: strategies compared and sub-trees.** TQS: Tétanos Quick Stick; TP: True Positive; FP: False Negative; TN: True Negative; FN: False Negative **(A)** True Positive sub-tree; **(B)** True Negative sub-tree; **(C)** False Positive sub-tree; **(D)** False Negative sub-tree; *For non-tetanus-prone wound; # For tetanus-prone wound.

We developed a decision-tree model that simulates the trajectory of a cohort of adult patients seeking care in EDs for a wound during the year 2012 in France. Each patient's trajectory incorporates wound type, tetanus immunization status diagnosis, administration of unscheduled tetanus prophylaxis according to immunization status, and the risk for tetanus and related survival with or without sequelae (Figure 1). Several parameters used in the model (immunization status, unscheduled vaccination guidelines, tetanus incidence) vary with age; we therefore stratified our adult study population into two age groups: 18-64 years old and ≥65 years. Since tetanus incidence and prophylaxis guidelines differ according to the type of wound, we also stratified the study population into patients with and without tetanus-prone wounds. The model was developed and analyzed using TreeAge Pro 2012 (TreeAge Software, Inc., Williamstown, MA).

### Strategies

Two strategies to diagnose the tetanus immunization status were compared and applied each on the entire hypothetical cohort of patients considered in the analysis.

#### (i) “Medical Interview” strategy (current practice)

The “Medical Interview” is illustrated in Figure 1. Patients were first asked whether they had their vaccination card. If they did, this card showed whether their last booster was up to date or not. If they did not, they are asked about their tetanus vaccination history in a medical interview. If they reported “No or unknown complete primary vaccination”, based on French guidelines, they were given a booster and a human tetanus immunoglobulin (TIG) injection in the case of tetanus-prone wounds, and only a booster in the case of no tetanus-prone wounds. If the patients asserted having received a “Complete primary vaccination”, they were asked if their boosters were up to date. Those replying yes received no prophylaxis; those replying no received a booster and a TIG injection in the case of tetanus-prone wounds and only a booster in the case of no tetanus-prone wounds.

#### (ii) “TQS” strategy

The “TQS” strategy is illustrated in Figure 1. As in the “Medical Interview” strategy, patients were first asked about their vaccination card. Patients without a vaccination card were systematically screened with a TQS. A positive test classified the patient as immune (i.e. with serum anti-tetanus antibody above 0.1 IU/mL of blood) [2]. Immune patients received no prophylaxis. A negative test classified the patient as non-immune (i.e. serum anti-tetanus antibody under the protective threshold). Non-immune patients received unscheduled tetanus prophylaxis like patients in the “Medical Interview” strategy.

### Outcomes

Based on this model, we estimated for each strategy the proportion of patients correctly and incorrectly diagnosed, the number of tetanus cases per year, and the cohort's life expectancy. We also assessed overall costs in order to calculate the incremental cost-effectiveness ratio (ICER) of the “TQS” strategy compared with current practice [10].

Since wound type determines ED care and has an impact on tetanus risk, we conducted a sub-analysis on tetanus-prone and non-tetanus-prone wounds. Because patients aged ≥65 years are less immune against tetanus, we also conducted a specific analysis in this population [11].

### Key assumptions

Key simplifying assumptions in this model included the following:

1. If patients came to the ED with a vaccination card, the health care professional considered the card as written evidence of the patient's true immunization status.
2. We considered that patients who were not immune and who received appropriate tetanus prophylaxis would not develop tetanus.

### Data and sources

Model input variable parameters were mainly estimated using national observational data and, when not available, through an extensive review of the international literature (Table 2).

**Table 2:** **Model parameters base case values and ranges used in the sensitivity analyses**

Parameter	Base case value	Min	Max	Source
Annual number of patients ≥18 years coming to French EDs	12,757,000	12,223,000	13,243,000	[12]
Cohort size (wounded patients ≥18 years coming to the French EDs)	1,658,000	1,589,000	1,722,000	Calculus based on [13] and [12]
Pr wound│ED consultation	13.0%	10.0%	15.0%	[13]
Pr tetanus-prone wound│wound	31.0%	18.1%	77.3%	[5],[6],[14]-[17]
Pr highly tetanus-prone wound│wound	4.9%			[6]
Pr patients ≥65 years in the cohort	16.6%	10.0%	20.0%	[8],[12]
Pr men in the cohort	70.3%	45.7%	75.0%	[5],[6],[8],[15],[16]
Life expectancy	(Pr men in the cohort ×Average men's life expectancy of the age group) + ((1- Pr men in the cohort)×Average women's life expectancy of the age group)			
18 to 64 years	40.9	38.9	44.7	[18]
≥65 years	9.1	8.4	10.4	
Pr vaccination card	11.9%	0.0%	19.6%	[5],[14],[16]
Medical interview sensitivity	62.0%	38.0%	65.0%	[4]-[8]
Medical interview specificity	79.0%	66.0%	96.0%	[4]-[8]
TQS sensitivity	69.0%	55.0%	96.0%	[4]-[8],[19],[20]
TQS specificity	98.0%	87.2%	100.0%	[4]-[8],[19],[20]
Seroprevalence (≥0.1 IU/mL)				
18 to 64 years	94.6%	84.0%	96.6%	[5]
≥65 years	76.6%	72.2%	83.3%	[21]
Pr of being up-to-date with booster shots				
18 and 64 years (last booster less than 20 years ago)	71.2%	70.0%	81.0%	[22]
≥65 years (last booster less than 10 years ago)	44.0%	44.0%	77.0%	[11]
Pr of booster being assessed as up-to-date	(Medical interview sensitivity×Pr up-to-date with boosters) + ((1-Medical interview specificity)			
	×(1-Pr up-to-date with boosters))			
18 to 64 years	50.2%			
≥65 years	39.0%			
Pr positive TQS	(TQS sensitivity×Seroprevalence) + ((1-TQS specificity)×(1-Seroprevalence))			
18 and 64 years	65.7%			
≥65 years	53.3%			
Pr of patient reporting having a complete primary vaccination	69.9%	71.5%	50.4%	[5],[15]
Medical interview PPV	Pr up-to-date with boosters×Medical interview sensitivity/((Pr up-to-date with boosters×Medical interview sensitivity) + ((1-Pr up-to-date with boosters)×(1-Medical interview specificity)))			
18 to 64 years	88.0%			
≥65 years	69.9%			
Medical interview NPV	(1-Pr up-to-date with boosters)×Medical interview specificity/(((1-Pr up-to-date with boosters)			
	×Medical interview specificity) + (Pr up-to-date with boosters×(1-Medical interview sensitivity)))			
18 to 64 years	45.7%			
≥65 years	72.6%			
TQS PPV	Seroprevalence×TQS sensitivity/((Seroprevalence×TQS sensitivity) + ((1-Seroprevalence)×(1-TQS specificity)))			
18 to 64 years	99.8%			
≥65 years	99.1%			
TQS NPV	(1-Seroprevalence)×TQS specificity/(((1-Seroprevalence)×TQS specificity ) + (Seroprevalence×(1-TQS sensitivity)))			
18 to 64 years	15.3%			
≥65 years	49.1%			
TIG relative risk on tetanus occurrence	0			Institut de veille sanitaire expert assumption
Tetanus vaccine relative risk on tetanus occurrence	1			Institut de veille sanitaire expert assumption
Pr hospitalization│tetanus case	100%			[23]-[26]
Pr death│tetanus case				
<70 years	10.0%	0%	100.0%	[23]-[26]
≥70 years	27.2%	0%	42.0%	[23]-[26]
Pr sequelae│surviving tetanus case	31.6%	18.8%	50.0%	[23]-[26]
c TQS (€ 2012)	€ 4.7	€ 4	€ 5	[9],[17]
c tetanus vaccine (Revaxis®: 0.5 mL syringe) (€ 2012)	€ 10.0	€ 3	€ 15	[27]
c human TIG (Gammatetanos®: 250 IU/2 mL syringe) (€ 2012)	€ 34,9	€ 30	€ 40	[27]
c hospitalization tetanus case (€ 2012)	€ 209,000	€ 150,000	€ 250,000	[28]
c sequelae of a tetanus case (€ 2012)	€ 5,663	€ 5,000	€ 6,000	[28]

Pr: probability; c: cost; │: among (in case of a conditional probabilities); ED: Emergency Department; TQS: Tétanos Quick Stick; TIG: Tetanus immunoglobulins; PPV: Positive predictive value; NPV: Negative predictive value; GP: General practitioner.

Our study population was an annual cohort of wounded adults seeking care in an ED. These correspond to 13% of the 12.7 million adults annually seeking care in French EDs, 16.6% of whom were aged ≥65 years, and 70.3% were male [5],[8],[12],[13]. Children were not considered in this analysis because data on TQS and medical interview performance are lacking in this population. Based on Colombet et al. data, 31% of the patients had a tetanus-prone-wound [5].

The true immunization rate of patients aged 18-64 years was estimated to be 94.6% based on ELISA test results in the Colombet et al. study (see Additional file 1: Technichal Appendix) [5]. Seroprevalence in patients aged ≥65 years was estimated as 76.6% using data from a study of vaccination coverage in this population by the Institut de Veille Sanitaire, which is the French Institute for Public Health Surveillance.

The probability of having a vaccination card when coming to the ED was estimated as 12% [5]. The performances of the two screening strategies in defining the immunization status of patients were estimated using the findings of Colombet et al. [5]. The sensitivity and specificity of TQS testing of blood samples in ED conditions were estimated to be 69% and 98%, respectively [5]. Based on the same data, medical interview sensitivity and specificity were estimated as 62% and 79%, respectively.

Tetanus incidence was estimated based on age, the type of wound, and the administration or not of unscheduled tetanus prophylaxis. Tetanus incidence rates were calculated by age group and type of wound based on national observational data between 2000 and 2011 (see Additional file 1: Technical Appendix).

Specific tetanus incidence rates obtained were: for patients between 18-64 years, 0.3 and 1.6 cases per million for non-tetanus-prone and tetanus-prone wounds respectively; and 8.8 and 41 cases per million for patients aged ≥65 years (see Additional file 1: Technical appendix). In patients in whom tetanus occurred, we considered that the probability of being hospitalized was 1. Mortality and the probability of long-term sequelae were estimated using data on the surveillance of tetanus from the French Institut de Veille Sanitaire [23]-[26].

### Costs

The estimated cost of the TQS was €4.7 according to Lesimple et al. [9]. No TQS administration cost was considered, assuming that costs associated with the working time spent performing the test would be roughly equal to the time needed to conduct the medical interview. Treatment costs included tetravalent booster vaccine (Revaxis®, Sanofi Pasteur MSD: €10) and TIG (Gammatetanos®, Laboratoire LFB Biomédicaments: €34.9) and were obtained from the Vidal (French equivalent of the Physicians' Desk Reference), an online database of information on healthcare products [27]. The cost of a tetanus case was estimated using national hospitalization statistics (= €209,000) [28]. Costs related to tetanus sequelae were assessed by taking into account stays in the follow-up care and rehabilitation departments also using national hospitalization statistics and were estimated as €5,390 [28].

Outpatient costs related to tetanus cases were not included since in this analysis in this analysis since they were considered to be negligible when compared to hospitalization costs. In cost calculations, we adopted a societal perspective. We only included direct and differential medical costs between the strategies compared which were all supported by the French Health Insurance (in 2012 euros). The tetanus incidence is very low in France and the study perspective was fixed at one year [1]. Consequently, productivity cost were considered as negligible and not included. Since the study does not include human material or human data, because only published data from a literature review were used, the approval of an ethics committee was not needed.

### Sensitivity analysis

In addition to a sub-analysis based on the nature of the wound (tetanus-prone or not) and the patient's age, one-way sensitivity analyses were performed for all probabilities and costs, to evaluate the robustness of our results and to explore the impact that parameter uncertainties. In those analyses, we used estimates for input variables found in the medical literature but not used in the base case analysis. When input variables were only from one study, a plausible range of values was built based on assumptions. For the parameters identified in the one-way sensitivity analysis as having a high impact on results, a threshold analysis was performed to determine at which values outside plausible ranges the choice of optimal strategy could be changed. We also considered and explored several scenarios in our sensitivity analysis. We considered cases where a monovalent vaccine or equine immunoglobulins were administered (see Additional file 1: Technical Appendix).

## Results

Under current practice, our model predicted approximately one tetanus case per year among those who sought medical care in the ED for a wound. This correlated well with the observed number of tetanus cases per year in France caused by wound infection and that would have led to an ED consultation [23]-[26].

### Base-case analysis

In the present context, for 1,658,000 patients presenting at the ED with wounds, the use of the “TQS” strategy resulted in 0.02 tetanus cases per year compared with 0.41 cases per year for “Medical Interview” (Table 3). The number of tetanus cases with each strategy was thus low and comparable. We will therefore only focus on costs and consider that the current analysis is a cost-minimization rather than a cost-effectiveness analysis.

**Table 3:** **Effectiveness and cost of tetanus immunity diagnostic strategies in wounded patients in French ED** ^**a,b**^

Cohort	Strategies	Tetanus case	Effectiveness (LY)	Vaccine dose	TIG dose	TQS cost	Prophylaxis cost	Total cost
All wounded patients	Medical Interview	0.41	58,658,086.40	1,033,000	320,000	-	€ 21,478,000	€ 21,564,000
N =1,658,000	TQS	0.02	58,658,087.40	601,000	186,000	€ 6,866,000	€ 12,490,000	€ 19,361,000
	Δ^C^	-0.39	1	-432,000	-134,000	€ 6,866,000	-€ 8,988,000	-€ 2,203,000
Patients with a	Medical Interview	0.13	40,494,923.00	713,000	-	-	€ 7,118,000	€ 7,145,000
Non-tetanus-prone wound	TQS	0.01	40,494,923.30	415,000	-	€ 4,740,000	€ 4,139,000	€ 8,881,000
N =1,145,000	Δ	-0.13	0.3	-298,000	-	€ 4,740,000	-€ 2,979,000	€ 1,736,000
Patients with a	Medical Interview	0.28	18,163,163.40	320,000	320,000	-	€ 14,360,000	€ 14,419,000
Tetanus-prone-wound	TQS	0.02	18,163,164.10	186,000	186,000	€ 2,126,000	€ 8,351,000	€ 10,480,000
N =513,000	Δ	-0.26	0.7	-134,000	-134,000	€ 2,126,000	-€ 6,009,000	-€ 3 939,000
Patients 18-64 years	Medical Interview	0.04	56,180,160.90	838,000	260,000		€ 17,048,000	€ 17,438,000
N =1,383,000	TQS	0	56,180,161.00	469,000	145,000	€ 5,726,000	€ 9,755,000	€ 15,482,000
	Δ	-0.04	0.10	-369,000	-115,000	€ 5,726,000	-€ 7,293,000	-€ 1,956,000
Patients of 65	Medical Interview	0.37	2,477,925.50	195,000	60,000	-	€ 4,430,000	€ 4,126,000
Years and over	TQS	0.02	2,477,926.40	132,000	41,000	€ 1,140,000	€ 2,735,000	€ 3,879,000
N =275,000	Δ	-0.35	0.9	-63,000	-19,000	€ 1,140,000	-€ 1,695,000	-€ 247,000

ED: Emergency departments; LY: Life years; LYG: Life Years Gained; TQS: Tétanos Quick Sticks; TIG: Human tetanus immunoglobulins; N: Cohort size.

^a^Only wounded patients over 18 years coming in 2012.

^b^The number of tetanus cases with each strategy was low and comparable; differences in effectiveness was negligible. We therefore only focus on costs and consider that the current analysis is a cost-minimization rather than a cost-effectiveness analysis.

^c^Δ = Defined as the delta of the transition from the “Medical Interview” strategy to the “TQS” strategy.

The overall “TQS” strategy was less costly than “Medical Interview”: €19,361,000 compared with €21,564,000. Increased costs incurred by TQS use (€6,866,000) were offset by reduction of unnecessary prophylaxis (€8,988,000): 42% decreases in booster shots and TIG doses. The average cost per patient was: €13 with “Medical Interview” vs. €11.7 with “TQS” for the overall cohort.

### Sub-analysis results

The two sub-groups where the TQS use generated the greatest savings were the tetanus-prone wounds (€3,939,000) and the 18-64 years old patients (€1,956,000). For patients aged ≥65 years, use of TQS also decreased overall costs, but to a lesser extent (€247,000). However, for the non-tetanus-prone wounds cohort, the “TQS” strategy cost more than “Medical Interview” while being more effective. The transition from current practice to the “TQS” strategy however cost €5,292,000/life years gained (LYG). The average cost per patient with “Medical Interview” vs. “TQS” was: €6.2” vs. €7.8 for non-tetanus-prone wounds; €28.9 vs. €21 for tetanus-prone wounds; €126.1 vs. €112 for patients between 18-64 years of age and €15 vs. €14.1 for patients aged ≥65 years.

### Sensitivity analysis

#### One way

**Figure 2:**
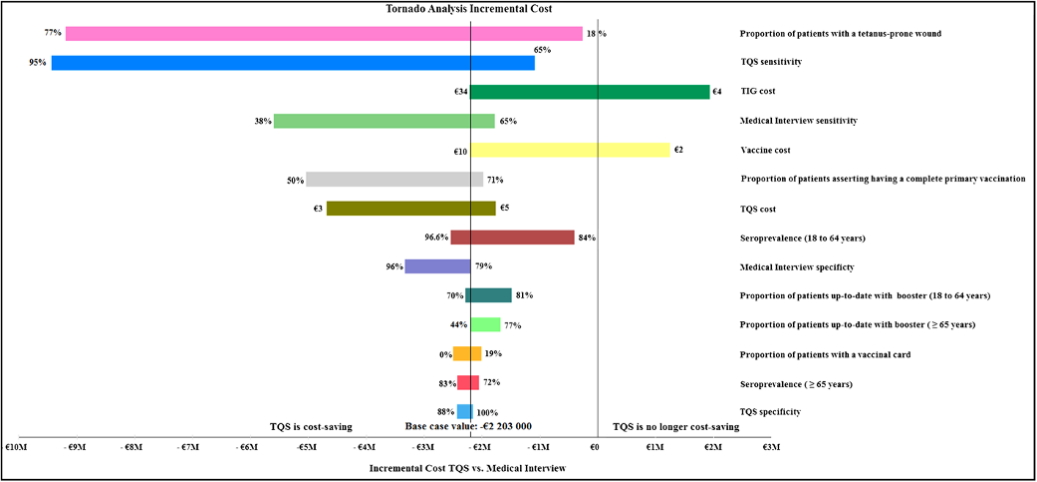
**Tornado analysis on strategy incremental costs: incremental cost of the “TQS” strategy compared with the “Medical Interview” strategy (in 2012 Million euros).** TQS: Tétanos Quick Stick; TIG: Human tetanus immunoglobulins.

A one-way sensitivity analysis on cost and effectiveness was performed for each variable across their range of plausible values shown in Table 2. Figure 2 shows the parameters values for which the model was most sensitive to the global costs of the strategies' global costs. The proportion of tetanus-prone wounds in the cohort and TQS sensitivity were the parameters whose variation led to the highest change in costs, but without inversion of the preferred strategy. Indeed, a decrease in prophylaxis costs could change the base-case conclusion. For a vaccine cost below €4.9 (vs. €10 in the base case), and a TIG cost below €18.5 (vs. € 34.9 in the base case), “TQS” was no longer cost-saving compared with current practice.

#### Threshold analysis

Results of the threshold analysis showed that for a TQS sensitivity under 61.2% (vs. 69% in the base case), or a TQS cost over €6.2 (vs. €4.7 in the base case), the TQS cost was no longer cost-saving compared with the “Medical Interview” strategy.

#### Alternative scenarios

When we considered that patients were treated with equine TIG (which cost €4.1 vs. €34.9 for the human TIG in the base case), “TQS” was no longer cost-saving and was associated with an ICER of 875,000 €/LYG. Similar results were observed when we explored the case where a monovalent vaccine was administered (€2.82 vs. €10 for the tetravalent vaccine used in the base case) with an ICER of 1,887,000€/LYG (Additional file 1: Technical Appendix). In every other scenarios and sensitivity analysis performed where the “TQS” cost was higher than “Medical Interview”, it never appeared to be cost-effective.

## Discussion

We found that in adult patients presenting to the ED with wounds, for comparable effectiveness “TQS” was less expensive than a strategy based on medical interview. The difference in costs was mainly due to the decrease in unnecessary tetanus prophylaxis achieved because of the higher sensitivity of TQS in identifying protected patients who do not need prophylaxis. Sub-analyses showed that the “TQS” strategy is especially cost-saving in patients with tetanus-prone wounds, where costly TIG injections are recommended, and for patients with a low tetanus seroprevalence (the elderly). However the TQS cost was higher than current practice for non-tetanus prone wounds or when lower prophylaxis costs were explored; and in those cases TQS was associated with high ICER. This was due to the fact the number of cases adverted compared to current practice was always negligible due to low tetanus incidence rates. Therefore, the very small gap in effectiveness between the strategies lead to extremely high ICERs that were never considered cost-effective in the French context (>3 × French GPD = €90,000).

While few clinical studies have been conducted in other countries, this study is to our knowledge the first model-based study to evaluate the cost-effectiveness of the TQS in France. This study design allows us to simulate the impact of TQS use as well as several alternative scenarios of wound management. Results on effectiveness obtained with our model converge to those reported by French institutions (<1 case of tetanus per year among patients who present to the ED vs. 0.45 in our model for the “Medical Interview” strategy). This illustrates the internal validity of our results.

Our results are consistent with previous epidemiological studies. For example, Hatamabadi et al. found the use of TQS in Tehran, Iran, to be cost-saving for emergency patients with tetanus-prone wounds (average cost per patient €12.1 with “Medical Interview” vs. €9.5 with “TQS”), but not for patients with non-tetanus-prone wounds (€0.1 with “Medical Interview” vs. €4 with the “TQS”) [7]. In an assessment of test efficiency in five hospitals in three regions of Belgium (Brussels, Flanders and Wallonia), Stubbe et al. found that TQS use was cost-saving for tetanus-prone wounds for all age groups (€10.6/patient with the “TQS” vs. €11.3 with “Medical Interview”) [14]. However, they found that TQS use did not lead to cost-saving for non-tetanus-prone wounds (€7.3/patient with the “TQS” vs. €3.9/patient with “Medical Interview”) [14].

Our results were sensitive to prophylaxis costs. For example, for a TIG cost under €18.5 or vaccine cost under €4.9, the “Medical Interview” strategy became less expensive than “TQS”. These values are above the cost of equine TIG and of monovalent vaccine, which suggests that TQS use would not be cost-saving if tetanus prophylaxis was solely based on those components. Variation of TQS sensitivity and cost could also change the results. However, their threshold values were outside plausible ranges found in the literature, and very far from values considered in our base case analysis, which were conservative when considering the “Medical Interview” strategy. This illustrates the robustness of our results.

Our study has several limitations. First, our results are valid for the specific French ED setting, wound care management and costs. However, given that wound management is quite comparable, at least in other European countries, our results can probably be extrapolated to other settings. Second, data used for the model came from various studies and some may not represent the French context. However, model inputs were varied over a wide range and our results were robust to these variations. Third, for reasons of feasibility and data availability, only two wound categories were considered. The third category of wounds in French clinical guidelines for tetanus prophylaxis was not considered (Table 1). We included this category (which seems to represent only 4.9% of all types of wounds observed in EDs [6]) in the “tetanus-prone wound” category. Because patients with this third type of wound must receive two TIG injections, this could have resulted in underestimation of the number of TIG injections prescribed in our model. Consequently, this might have led to underestimation of the reduction of prophylaxis costs generated by use of TQS, which further strengthens our results. The same would have been observed if primary vaccination follow-up had been considered; indeed, due to the higher specificity of the TQS, fewer patients incorrectly identified as not having received primary vaccination would have been sent to the general practitioner.

Although the impact of those tests have been evaluated in high income countries, their use could be highly relevant in poorer countries with a higher tetanus incidence due to a lack of tetanus immunity in wounded patients.. In such settings, TQS use could minimize tetanus prophylaxis costs, but more importantly maximize effectiveness in terms of life years gained, due to its high specificity in the detection of unprotected patients.

## Conclusions

In conclusion, in patients seeking care for a wound in French EDs, TQS use is cost-saving compared with the “Medical Interview” strategy. The TQS is especially cost-saving in patients with tetanus-prone wounds, and to a lesser extent in patients aged ≥65 years. Therefore, its use should be specifically recommended in this population.

## Additional file

## Electronic supplementary material

Additional file 1: Technical Appendix. We assessed the effectiveness and cost of TQS use in French emergency departments (EDs) in patients seeking care for a wound, compared with the medical interview regarding vaccination history. We developed a decision-tree model that retraces clinical practice in the ED and includes screening for immunity to tetanus in wounded patients, conditional prophylaxis administration, and risk of tetanus occurrence. Data used as input in the model were found through an extensive literature review. In the Technical Appendix we describe in detail the sources of the probabilities and costs selected as parameters. We explain the methods and provide the formula used to estimate the cohort life expectancy and the patient's tetanus immunity and its identification by the two diagnostic methods compared. We also report in detail how we built incidence rates in non-protected patients who were incorrectly diagnosed, according to their age and type of wound. Finally, we present additional results of the sensitivity analyses conducted as part of this study. (DOCX 41 KB)

Below are the links to the authors’ original submitted files for images.

Authors’ original file for figure 1

Authors’ original file for figure 2

## Abbreviations

c: Cost
ED: Emergency Department
FN: False Negative
FP: False Negative
GP: General practitioner
ICER: Incremental cost effectiveness ratio
LY: Life years
LYG: Life Years Gained
N: Cohort size
NPV: Negative predictive value
Pr: Probability
PPV: Positive predictive value
(TQS): Tétanos Quick Stick®
TIG: Tetanus immunoglobulin
TN: True Negative
TP: True Positive
│: Among (in case of a conditional probabilities)
