# Supplementary material for: Effectiveness and cost of quick diagnostic tests to determine tetanus immunity in patients with a wound in french emergency departments
Source: BMC Infect Dis. 2014 Nov 19;14:603. doi: 10.1186/s12879-014-0603-3 (PMC4246690; doi:10.1186/s12879-014-0603-3)
Supplement: Supplementary file 1 — Additional file 1: Technical Appendix. We assessed the effectiveness and cost of TQS use in French emergency departments (EDs) in patients seeking care for a wound, compared with the medical interview regarding vaccination history. We developed a decision-tree model that retraces clinical practice in the ED and includes screening for immunity to tetanus in wounded patients, conditional prophylaxis administration, and risk of tetanus occurrence. Data used as input in the model were found through an extensive literature review. In the Technical Appendix we describe in detail the sources of the probabilities and costs selected as parameters. We explain the methods and provide the formula used to estimate the cohort life expectancy and the patient's tetanus immunity and its identification by the two diagnostic methods compared. We also report in detail how we built incidence rates in non-protected patients who were incorrectly diagnosed, according to their age and type of wound. Finally, we present additional results of the sensitivity analyses conducted as part of this study. (DOCX 41 KB) [file 12879_2014_603_MOESM1_ESM.docx]

**Additional file 1: Technical Appendix**

**Effectiveness and Cost of Quick Diagnostic Tests to Determine Tetanus Immunity in Patients with a Wound in French Emergency Departments**

Dieynaba S. N’Diaye,

Michaël Schwarzinger,

Dorothée Obach,

Julien Poissy,

Sophie Matheron,

Enrique Casalino,

Yazdan Yazdanpanah.

**INTRODUCTION**

We assessed the effectiveness and cost of TQS use in French emergency departments (EDs) in patients seeking care for a wound, compared with the medical interview regarding vaccination history. We developed a decision-tree model that retraces clinical practice in the ED and includes screening for immunity to tetanus in wounded patients, conditional prophylaxis administration, and risk of tetanus occurrence.

Data used as input in the model were found through an extensive literature review. In this Technical Appendix we describe in detail the sources of the probabilities and costs selected as parameters. We explain the methods and provide the formula used to estimate the cohort life expectancy and the patient’s tetanus immunity and its identification by the two diagnostic methods compared. We also report in detail how we built incidence rates in non-protected patients who were incorrectly diagnosed, according to their age and type of wound. Finally, we present additional results of the sensitivity analyses conducted as part of this study.

**METHODS**

**Input Data**

**Tetanus immunity**

The true immunization rate of patients aged between 18 and 59 years was estimated based on ELISA test results in the Colombet et al. study, which was conducted in a French multicenter population of patients aged between 18 and 59 years old seeking medical attention for a wound in French EDs [1]. The study reported a 94.6% seroprotection rate with a serum tetanus antitoxin level above 0.1 IU/mL, which WHO guidelines consider protective [2]. Seroprevalence in patients aged ≥65 years was estimated as 76.6% using data from a study of vaccination coverage in this population by the French Institut de Veille Sanitaire. The probability of being up-to-date with boosters was 71.2% for the French population between 18 and 64 years (less than 20 years since the last booster) and 44.0% for those aged ≥65 years (less than 10 years since the last booster). These values are from studies conducted by the French Institut de Veille Sanitaire [3, 4]

**Patient screening**

The probability of being identified as protected against tetanus or not, and positive and negative predictive values were calculated based on the sensitivity and specificity of each diagnostic method. Also determined were the probability of being up-to-date with booster shots for the medical interview and seroprevalence for the TQS (formula shown Table A.1).

**Tetanus incidence**

Tetanus incidence was estimated based on age, the type of wound, and the administration or not of unscheduled tetanus prophylaxis. Tetanus incidence rates were calculated by age group and type of wound based on national observational data for 2000 to 2011.

***Numerator***

The number of tetanus cases was estimated by age based on mandatory disease notification data published by the Institut de Veille Sanitaire between 2000 and 2011 [5-8]. These data indicated an annual average of 3 cases in patients aged <70 years, and 14 cases in patients aged ≥70 years. The estimated 64.5% exhaustiveness of reporting was included in our calculation [5-8].

Since our study population was ED patients, we focused only on tetanus cases involving an emergency consultation. We assumed that only patients with acute open wounds came to the ED (as opposed to those with chronic wounds or with an unidentified cutaneous portal of entry, who are more likely to go to their general practitioners). Morbidity and Mortality Weekly Report considers that 65.9% of tetanus cases are due to acute wounds, taking into account the fact that not all patients with acute wounds present to the ED ([9]). According to the same American data, only 36.5% of tetanus patients presenting with an acute open wound come to the ED ([9]. The proportion of tetanus cases due to tetanus-prone wounds was estimated as 67.7% [5-8].

***Denominator***

Our study population was 1 658 000 adult patients, 16.6% of whom were used to calculate specific incidence rates for patients aged ≥65 years, and 31% for tetanus-prone wounds [1, 10-12].

Using the formula presented in Table A.2, we calculated specific tetanus incidence rates: for patients between 18 and 64 years, 0.3 and 1.6 cases per million for non-tetanus-prone and tetanus-prone wounds, respectively; and 8.8 and 41 cases per million for patients aged ≥65 years.

**Outcomes**

In patients in whom tetanus occurred, we considered that the probability of being hospitalized was 1. Mortality and the probability of long-term sequelae were estimated using data on the surveillance of tetanus from the Institut de Veille Sanitaire [5-8].

The cohort’s average life expectancy at entry in the model was calculated according to sex and the average age of patients seeking care in French EDs (see formula Table A.1). It was based on 2011 data from the French National Institute of Statistics and Economic Studies and estimated as 40.9 and 9.1 years for patients aged between 18 and 64 and ≥65 years, respectively ([http://www.insee.fr/Population-data-2001](http://www.insee.fr/fr/themes/detail.asp?ref_id=bilan-demo&reg_id=0&page=donnees-detaillees/bilan-demo/pop_age3.htm)). Life expectancy was at 0 when lethal tetanus case occurred and remained the same if not.

**Costs**

Hospital costs related to tetanus cases were estimated using national hospitalization statistics [13]. The diagnosis related group in which most of the tetanus cases were found was 18M104 corresponding to “Severe infectious diseases Level 4”. Its cost was modified by adjusting costs related to the intensive care unit as if all tetanus cases were admitted to this unit. The average period of time in intensive care was estimated as 42 days. The cost of the modified Diagnosis Related Group was estimated as €209,000.

Costs associated with tetanus sequelae were assessed by taking into account stays in the follow-up care and rehabilitation departments, based on the diagnosis related group 40A22 “Rehabilitation of adult patients” for which the estimated average total period was 17 days [13]. The estimated cost of a one-day stay in these departments was €317, so the overall cost was €5391 (=317 x 17 = 5391) per stay [13].

**Sensitivity analysis**

**Alternative scenarios**

We also explored several scenarios in our sensitivity analysis. We considered another case where a monovalent vaccine was administered (Vaccin Tétanique Pasteur®, €2.8), instead of the tetravalent vaccine used in the base case (Revaxis®, €10). We also considered an alternative where patients were treated with equine TIG (Immunoglobulines Équines Tétaniques Pasteur®, €2.8) instead of human TIG in the base case (Gammatetanos®, €34.9) [14].

**RESULTS**

**Alternative scenarios**

We found that when patients were treated with equine TIG, the “TQS” strategy was no longer cost-saving compared with “Medical Interview”. Similar results were observed when we explored the case where a monovalent vaccine was administered. These results are shown in Table A.3 (Effectiveness and cost of tetanus immunity diagnostic strategies in wounded patients in French ED: scenario with a monovalent tetanus vaccine) and Table A.4. (Effectiveness and cost of tetanus immunity diagnostic strategies in wounded patients in French ED: scenario with an equine TIG)

**REFERENCES**

1. Colombet I, Saguez C, Sanson-Le Pors MJ, Coudert B, Chatellier G, Espinoza P: **Diagnosis of tetanus immunization status: multicenter assessment of a rapid biological test**. *Clin Diagn Lab Immunol* 2005, **12**(9):1057-1062.

2. Borrow R, Balmer P, Roper M: **The immunological basis for immunisation series. Module 3: Tetanus Update 2006**. *© World Health Organization 2007, Department of Immunization, Vaccines and Biologicals* 2007(21 March 2011).

3. Gergely A, Bechet S, Goujon C, Pelicot M, Van Der Vliet D, Simons de Fanti A: **La couverture vaccinale contre le tétanos, la poliomyélite et la diphtérie en 2006 dans une population âgée francilienne.** *Bull Epidémiol Hebd* Saint-Maurice: Institut de veille sanitaire 2008(9):61-64.

4. Guthmann JP, Fonteneau L, Antona A, Lévy-Bruhl D: **La couverture vaccinale diphtérie, tétanos, poliomyélite chez l'adulte en France : résultats de l'Enquête santé et protection sociale, 2002**. *Bull Epidemiol Hebd* Saint-Maurice: Institut de veille sanitaire 2007, **51-52**:441-445.

5. Antona D: **Le tetanos en France entre 2000 et 2001.** *Bull Epidemiol Hebd* Saint-Maurice: Institut de veille sanitaire 2002, **40**:197-199.

6. Antona D: **Le tétanos en France en 2002-2004**. *Bull Epidemiol Hebd* Saint-Maurice: Institut de veille sanitaire 2006, **7**:53-55.

7. Antona D: **Le tétanos en France 2005 et 2007**. *Bull Epidemiol Hebd* Saint-Maurice: Institut de veille sanitaire 2008, **30-31**:273-275.

8. Antona D: **Le tétanos en france en 2008-2011** *Bull Epidemiol Hebd* Saint-Maurice: Institut de veille sanitaire 2012(26: ):53-55.

9. **Tetanus surveillance --- United States, 2001-2008**. *MMWR Morb Mortal Wkly Rep* 2011, **60**(12):365-369.

10. Stubbe M, Swinnen R, Crusiaux A, Mascart F, Lheureux PE: **Seroprotection against tetanus in patients attending an emergency department in Belgium and evaluation of a bedside immunotest**. *Eur J Emerg Med* 2007, **14**(1):14-24.

11. Société Francophone de Médecine d’Urgence : SFMU: **Prise en charge des plaies aux urgences** In*.*: http://www.sfmu.org/documents/consensus/cc_plaies_longue.pdf; Copyright@SFMU 2005.

12. **Directorate for Research, Studies, Evaluation and Statistics (DREES) annual statistical survey on healthcare facilities from 2008 to 2012** [http://www.sae-diffusion.sante.gouv.fr/Collecte_2012/dwd_dwsgen3.aspx]

13. **The Agency for Information on Hospital Care (ATIH) 2010 costs database** [http://www.atih.sante.fr/index.php?id=0009200003FF]

14. **French Online Physicians’ Desk Reference database** [http://www.vidal.fr/]

**TABLES**

| **Parameter** | | **Formula and base case value obtained** | | | | | |
| --- | --- | --- | --- | --- | --- | --- | --- |
| Life expectancy | | (Pr men in the cohort *Average male life expectancy of the age group) + ((1- Pr men in the cohort)  *Average female life expectancy of the age group) | | | | | |
| 18 to 64 years | | 40.9 |  |  |  | | |
| ≥65 years | | 9.1 |  |  |  | | |
| Pr of booster being assessed as up-to-date | | (Medical interview sensitivity*Pr up-to-date with boosters)+((1-Medical interview specificity)*(1-Pr up-to-date with boosters)) | | | | | |
| 18 to 64 years | | 50.2% |  |  |  | | |
| ≥65 years | | 39.0% |  |  |  | | |
| Pr positive TQS | | (TQS sensitivity*Seroprevalence)+((1-TQS specificity )*(1-Seroprevalence)) | | | | | |
| 18 to 64 years | | 65.7% |  |  |  | | |
| ≥65 years | | 53.3% |  |  |  | | |
| Medical interview PPV | | Pr up-to-date with boosters*Medical interview sensitivity/((Pr up-to-date with boosters*Medical interview sensitivity)+((1-Pr up-to-date with boosters)*(1-Medical interview specificity))) | | | | | |
| 18 to 64 years | | 88.0% |  |  |  | | |
| ≥65 years | | 69.9% |  |  |  | | |
| Medical interview NPV | | (1-Pr up-to-date with boosters)*Medical interview specificity/(((1-Pr up-to-date with boosters)*Medical interview specificity)  + (Pr up-to-date with boosters*(1-Medical interview sensitivity))) | | | | | |
| 18 to 64 years | | 45.7% |  |  |  | | |
| ≥65 years | | 72.6% |  |  |  | | |
| TQS PPV | | Seroprevalence*TQS sensitivity/((Seroprevalence*TQS sensitivity)+((1-Seroprevalence)*(1-TQS specificity ))) | | | | | |
| 18 to 64 years | | 99.8% |  |  |  | | |
| ≥65 years | | 99.1% |  |  |  | | |
| TQS NPV | | (1-Seroprevalence)*TQS specificity /(((1-Seroprevalence)*TQS specificity )+(Seroprevalence*(1-TQS sensitivity))) | | | | | |
| 18 to 64 years | | 15.3% |  |  |  | | |
| ≥65 years | | 49.1% |  |  |  | | |
| Pr: probability; TQS: Tétanos Quick Stick; PPV: Positive predictive value; NPV: Negative predictive value | | | | | |  |  |
|  | | | | | |  |  |

**Table A1 - Formula used in the model**

| **Parameter** | **Base case value** | **Min** | **Max** | | **Source** |
| --- | --- | --- | --- | --- | --- |
| Cohort size (patients ≥18 years coming to French EDs because of a wound) | 1,658,000 | 1,589,000 | 1,722,000 | | Calculus based on [11] and[12] |
| Number of tetanus cases in France in 2000-2011 |  |  |  | |  |
| <70 years | 3 | 0 | 7 | | [5-8] |
| ≥70 years | 14 | 2 | 23 | | [5-8] |
| Exhaustiveness of mandatory reporting of tetanus cases | 64.5% | 60.0% | 66.0% | | [5-8] |
| Pr ED consultation for a wound│tetanus case | 36.5% | 30.0% | 40.0% | | [9] |
| Pr tetanus-prone wound│tetanus case | 67.7% | 53.6% | 100% | | [5-8] |
| Pr acute wound│tetanus case | 65.9% | 44.8% | 73.1% | | [5-8] |
| Pr tetanus-prone wound│wound | 31.0% | 18.1% | 77.3% | | [5-9] |
| Pr patients ≥65 years in the cohort | 16.6% | 10.0% | 20.0% | | [10, 12], |
| Tetanus incidence rates (per million) | (Annual number of tetanus case*Pr acute wound│tetanus *Pr type of wound│tetanus*Pr ED consultation for a wound│tetanus) /(Exhaustivity of tetanus declaration*Cohort size*Pr of patient of the age group)) | | | | |
| 18 to 64 years |  |  |  | |  |
| Non-tetanus-prone wound | 0.3 |  |  | |  |
| Tetanus-prone wound | 1.6 |  |  | |  |
| ≥65 years |  |  |  | |  |
| Non-tetanus-prone wound | 8.8 |  |  | |  |
| Tetanus-prone wound | 41.0 |  |  | |  |
| Pr: probability; │: among (in case of a conditional probabilities); ED: Emergency department | | | |  |  |

**Table A.2- Model parameters used to calculate the tetanus rate incidence**

**Table A.3 - Scenario with a monovalent tetanus vaccine ^a^**

| Cohort | Strategies | Tetanus | Life Years | Strategy | TQS | Prophylaxis | Vaccine | TIG |
| --- | --- | --- | --- | --- | --- | --- | --- | --- |
|  |  | case | (LY) | Cost | Cost | Cost | dose | dose |
| All wounded patients | Medical Interview | 0.41 | 58,658,086.4 | € 14,167,000 | - | € 14,081,000 | 1,033,000 | 320,000 |
| N = 1,658,000 | TQS | 0.02 | 58,658,087.4 | € 15,060,000 | € 6,866,000 | € 8,189,000 | 601,000 | 186,000 |
|  | ∆ | -0.39 | 1.0 | € 893,000 | € 6,866,000 | -€ 5,892,000 | -432,000 | -134,000 |
| ED: Emergency departments; LY: Life years; TQS: Tétanos Quick Sticks; TIG: Human tetanus immunoglobulins; N: Cohort size | | | | | | | | |
| ^a^ Effectiveness and cost of tetanus immunity diagnostic strategies in wounded adult patients presenting to French EDs in 2012.  ∆= Defined as the delta of the transition from the Medical Interview strategy to the TQS strategy | | | | | | | | |

**Table A.4 - Scenario with an equine TIG ^a^**

| Cohort | Strategies | Tetanus | Life Years | Strategy | TQS | Prophylaxis | Vaccine | TIG |
| --- | --- | --- | --- | --- | --- | --- | --- | --- |
|  |  | case | (LY) | Cost | Cost | Cost | dose | dose |
| All wounded patients | Medical Interview | 0.41 | 58,658,086.4 | € 11,721,000 | - | € 11,635,000 | 1,033,000 | 320,000 |
| N = 1,658,000 | TQS | 0.02 | 58,658,087.4 | € 13,637,000 | € 6,866,000 | € 6,766,000 | 601,000 | 186,000 |
|  | ∆ | -0.39 | 1.0 | € 1,916,000 | € 6,866,000 | -€ 4,869,000 | -432,000 | -134,000 |
| ED: Emergency departments; LY: Life years; TQS: Tétanos Quick Sticks; TIG: Human tetanus immunoglobulins; N: Cohort size  ^a^ Effectiveness and cost of tetanus immunity diagnostic strategies in wounded adult patients presenting to French EDs in 2012. | | | | | | | | |
| ∆= Defined as the delta of the transition from the Medical Interview strategy to the TQS strategy | | | | | | | | |
