# Supplementary figures and images for: Effectiveness and cost of quick diagnostic tests to determine tetanus immunity in patients with a wound in french emergency departments
Source: BMC Infect Dis. 2014 Nov 19;14:603. doi: 10.1186/s12879-014-0603-3 (PMC4246690; doi:10.1186/s12879-014-0603-3)

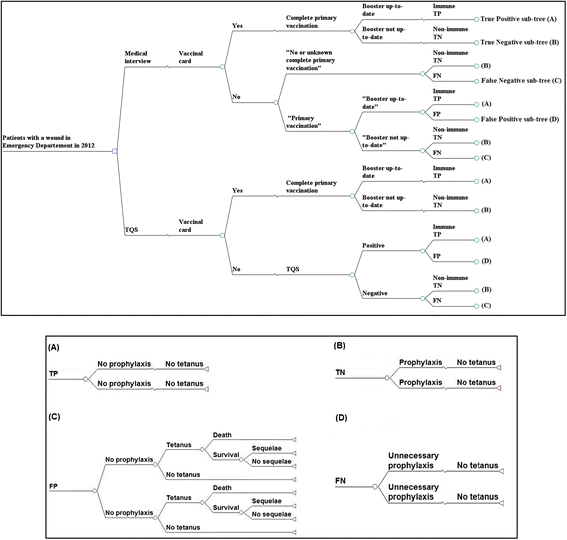

Supplement: Supplementary file 2 — Authors’ original file for figure 1 [file 12879_2014_603_MOESM2_ESM.gif]

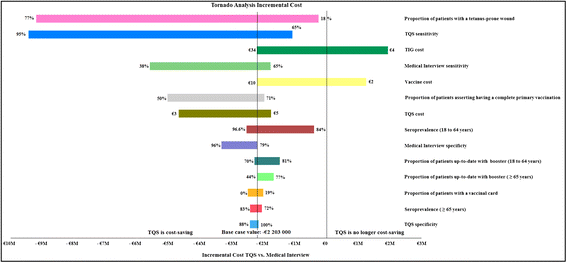

Supplement: Supplementary file 3 — Authors’ original file for figure 2 [file 12879_2014_603_MOESM3_ESM.gif]
